# Ultralow Power Wearable Heterosynapse with Photoelectric Synergistic Modulation

**DOI:** 10.1002/advs.201903480

**Published:** 2020-03-16

**Authors:** Tian‐Yu Wang, Jia‐Lin Meng, Zhen‐Yu He, Lin Chen, Hao Zhu, Qing‐Qing Sun, Shi‐Jin Ding, Peng Zhou, David Wei Zhang

**Affiliations:** ^1^ State Key Laboratory of ASIC and System School of Microelectronics Fudan University Shanghai 200433 China

**Keywords:** artificial heterosynapses, neuromorphic computing architectures, photoelectric synergistic modulation, synaptic devices, wearable electronics

## Abstract

Although the energy consumption of reported neuromorphic computing devices inspired by biological systems has become lower than traditional memory, it still remains greater than bio‐synapses (≈10 fJ per spike). Herein, a flexible MoS_2_‐based heterosynapse is designed with two modulation modes, an electronic mode and a photoexcited mode. A one‐step mechanical exfoliation method on flexible substrate and low‐temperature atomic layer deposition process compatible with flexible electronics are developed for fabricating wearable heterosynapses. With a pre‐spike of 100 ns, the synaptic device exhibits ultralow energy consumption of 18.3 aJ per spike in long‐term potentiation and 28.9 aJ per spike in long‐term depression. The ultrafast speed and ultralow power consumption provide a path for a neuromorphic computing system owning more excellent processing ability than the human brain. By adding optical modulation, a modulatory synapse is constructed to dynamically control correlations between pre‐ and post‐synapses and realize complex global neuromodulations. The novel wearable heterosynapse expands the accessible range of synaptic weights (ratio of facilitation ≈228%), providing an insight into the application of wearable 2D highly efficient neuromorphic computing architectures.

## Graphical abstract

A flexible MoS_2_‐based heterosynapse is designed with electronic and photoexcited modes. With an ultrafast pre‐spike of 100 ns, the synaptic device exhibits ultralow energy consumption of 18.3 aJ per spike in long‐term potentiation and 28.9 aJ per spike in long‐term depression. The ultrafast speed and ultralow power consumption pave the way for a computing system owning more excellent processing ability than the human brain.

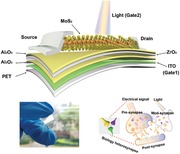

Conventional von Neumann architectures based on complementary metal‐oxide‐semiconductor (CMOS) circuits suffer from poor fault tolerance, low efficiency, complex algorithms, and huge energy consumption due to serial processing.^[^ ^1^, ^2^ ^]^ The human brain consisting of ≈86 billion neurons and the parallel processing neuron network has a power consumption of 20 W.^[^ ^3^ ^]^ IBM computers consume 2.9 MW to finish only a part of the same task.^[^ ^4^ ^]^ The basic units of neural circuitry for functional plasticity in biological brain are believed to be synapses, which consume 10 fJ per synaptic event. Previous reports have proposed various artificial synaptic devices for simulating long‐term and short‐term plasticity.^[^ ^5^, ^6^, ^7^, ^8^, ^9^ ^]^ Although the development of artificial synaptic architectures has achieved energy consumption down to the femtojoule level,^[^ ^10^ ^]^ it is hard to achieve lower levels due to the slow response time and high‐level post‐synaptic current (PSC). These problems lead to a bottleneck in reducing the power of synaptic weights update. Hence, designing an appropriate device with high‐speed program and low‐level current are necessary for solving energy consumption problems.

Most previous reports were limited to single devices with simple connections and pre‐ and post‐synaptic learning rules, such as excitatory post‐synaptic current (EPSC), inhibitory post‐synaptic current (IPSC), short‐term plasticity (STP), paired pulse facilitation/depression (PPF/PPD), spike time‐dependent plasticity (STDP), learning‐forgetting‐relearning behaviors etc.^[^ ^11^, ^12^, ^13^ ^]^ The global synapses in neural networks with interconnections to tens of thousands of other synapses for information transmission by neurotransmitters are fundamental in massive parallelism, but are usually overlooked. In contrast to homosynapse, heterosynapse is a basic part of simplified global neural network and have multi‐terminal configurations, including pre‐, post‐, and modulatory‐synapse (mod‐synapse).^[^ ^14^ ^]^ Heterosynapse plays key roles in biological functions, including long‐term memory for synaptic growth and associative learning.^[^ ^15^, ^16^ ^]^ Therefore, it is important to simulate synaptic features with responses to modulatory terminal. As a novel gate‐control mode, optical control could realize multi‐terminal neuromodulations in heterosynapse, with the advantages of reduced thermal loss and simple operating mode. To better understand the complex global neuromodulations in heterosynapse, optoelectronic materials should be meticulously selected for multi‐terminal synapses' construction.

In another aspect, 2D transition metal dichalcogenides (TMDCs) have attracted increasing attention as promising candidates for next‐generation flexible multifunctional electronics due to their superior mechanical flexibility, unique electrical, and optical properties.^[^ ^17^, ^18^, ^19^, ^20^ ^]^ For example, MoS_2_‐based multi‐bit memory exhibits photoelectronic non‐volatile characteristics with high on/off ratio (≈10^7^), reliable switching operation and stable retention (10^4^ s).^[^ ^21^ ^]^ Mechanical exfoliation, as a common method for obtaining 2D devices, was commonly used in silicon wafers with thermally grown SiO_2_ for easier determination of the number of layers.^[^ ^22^, ^23^, ^24^, ^25^ ^]^ However, devices required an additional transfer process from the silicon wafer to the flexible substrate for wearable electronics applications.^[^ ^26^ ^]^ The additional transfer step increases the difficulty of the fabrication process, reduces device performance and leads to rough interfaces between the devices and the flexible substrate. Besides, although atomic layer deposition (ALD) process could be used in high temperature and low temperature, the fabrication process usually needs high temperature (e.g., 300 °C) for high quality ALD film in most reports. The high temperature is not compatible with flexible substrate. Therefore, developing a feasible process to prepare flexible mechanically exfoliated 2D devices and proposing a reliable low temperature ALD process in film deposition on flexible substrate are essential.

In this paper, we demonstrate ultralow energy consumption (<30 aJ per spike for weight change) and ultrafast operation (100 ns) in a multi‐terminal artificial heterosynapse based on MoS_2_. To prepare this flexible device, a one‐step mechanical exfoliation method for a flexible substrate without additional transfer is proposed. The artificial synapse shows reliable synaptic weight modulation for over 2000 spikes with attojoule energy consumption (18.3 aJ per spike in long‐term potentiation (LTP) and 28.9 aJ per spike in long‐term depression (LTD)), which can be attributed to its operation speed (100 ns) and low conductance (≈1 nS). Heterosynaptic plasticity was emulated using photoelectric synergistic modulation with electrical and optical spikes together or sequentially. It is worth noting that the connection weights in heterosynapse can be enhanced from 350 to 800 pA (ratio of excitation ≈ 228%) with photoelectric synergistic modulation compared to electrical modulation, resulting in accessing a wider range of synaptic weights, thus improving dynamic plasticity. Furthermore, inhibition plasticity is weakened and the dynamic range of conductance states is decreased by combining light stimulation with electrical modulation. These comprehensive results pave the way for building ultralow power consumption neuron networks for neuromorphic computing using dual‐gated wearable artificial heterosynapse activated by electrical and light simulation.

The schematics of biological heterosynapse composed of the pre‐, post‐, and modulatory‐synapse are illustrated in **Figure** **1**a. Figure 1b illustrates the proposed multi‐gate device architecture based on a MoS_2_ channel and activated using electricity and light. The MoS_2_ channel was mechanically exfoliated from the bulk materials and then transferred directly to the PET substrate (Figure S1, Supporting Information). The high‐resolution transmission electron microscopy (HRTEM) image of the cross section of the channel and dielectric layers are shown in Figure 1c. The artificial heterosynapse was fabricated on a flexible polyethylene terephthalate (PET) substrate (Figure 1d). Physical characterizations, including EDX mapping images and line‐scan analysis of the cross section are shown in Figure S2, Supporting Information. A low temperature ALD process at 130 °C was carefully designed and proposed to obtain heterostructure of gate oxides, including Al_2_O_3_ (bottom layer)/ZrO_2_ (interlayer), and Al_2_O_3_ (top layer), acting as a blocking/trapping/tunneling layer, respectively. The topography of the flexible device substrate was measured through atomic force microscopy (AFM) in Figure 1e. The Raman spectrum of MoS_2_ on PET and silicon are shown in Figure 1f. The two characteristic peaks appeared around 382 and 406 cm^−1^, corresponding to the in‐plane (E^1^_2g_) and out‐of‐plane (A_1g_) vibration mode, respectively. The Raman peaks positons are a bit modified (≈2 cm^−1^), which may be induced from different strain of MoS_2_ on two substrates.

**Figure 1:**
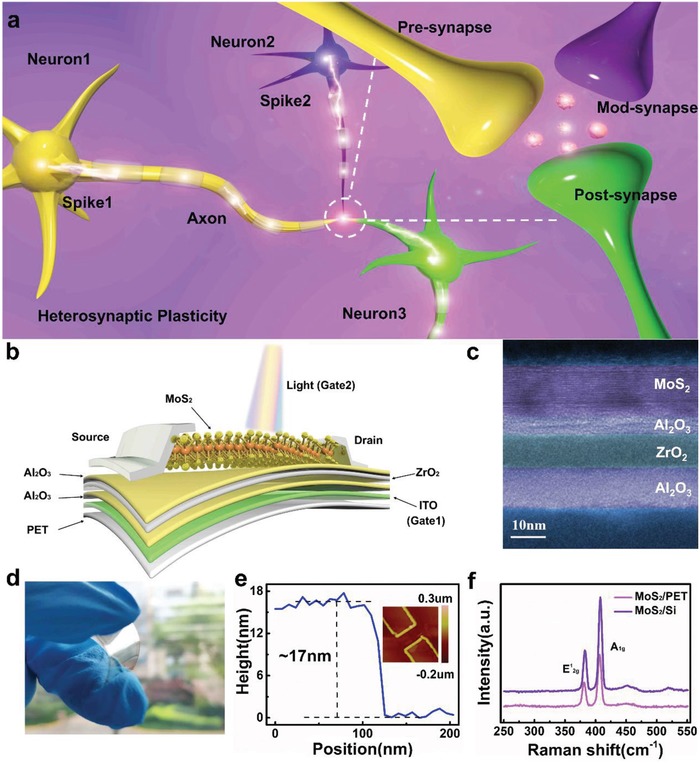
Schematic of biological heterosynapse and flexible artificial neuromorphic device. a) Process of transferring information in heterosynaptic system with pre‐synapse, mod‐synapse, and post‐synapse. b) The multi‐gated MoS_2_‐based flexible artificial heterosynapse with photoelectric dual modulation, in which MoS_2_ and ZrO_2_ serve as the semiconducting channel and trapping layer, respectively. The tunneling and blocking dielectrics are composed of Al_2_O_3_. c) HRTEM image of channel/tunneling layer/trapping layer/blocking layer in cross section. d) Photograph of MoS_2_ device under bending state. e) The channel height obtained from the AFM height image. Inset: AFM image of flexible device. f) Raman spectra of MoS_2_ flake on the substrate of silicon and Polyethylene terephthalate (PET).

The gate‐controlled transfer characteristics of the MoS_2_ memory were investigated in electrical mode (**Figure** **2**a), where the gate and drain are applied voltages, the source is grounded and the current of drain is monitored. Hysteresis appeared in the source–drain (*I*_DS_) versus control gate voltage (*V*_CG_) curve, while ohmic contacts are demonstrated in Figure S3, Supporting Information. A distinct clockwise hysteresis window of 7 V was obtained by dual‐sweeping *V*_CG_ from −7 to 7 V at a drain‐to‐source voltage (*V*_DS_) of 0.5 V, as shown in Figure 2b. The hysteresis window (Δ*V*_th_) could be enlarged gradually when the maximum control voltage (|*V*_CG,max_|) become larger (Figure 2c). The ultrahigh 10^6^ on/off ratio of |*V*_CG,max_| under 7 V allows the achievement of a wide range of modulated conductance states in emulating synaptic plasticity (Figure S3, Supporting Information). To determine the fastest programming speed, the transfer characteristics in the narrow range (−3V–3 V) after programming with different pulse width of *V*_CG_ are shown in Figure 2d, where the blue line shows the initialized state of memory after erase operation (−7 V/1 ms). Similarly, the device was initialized with a programming pulse (8.5 V/1 ms) and could be erased even when the width of *V*_CG_ was changed to 100 ns, as shown in Figure 2e. The ultrafast speed of synaptic device is important for constructing neuromorphic computing system with quicker processing speed than human brain, breaking the limitation of biological system.

**Figure 2:**
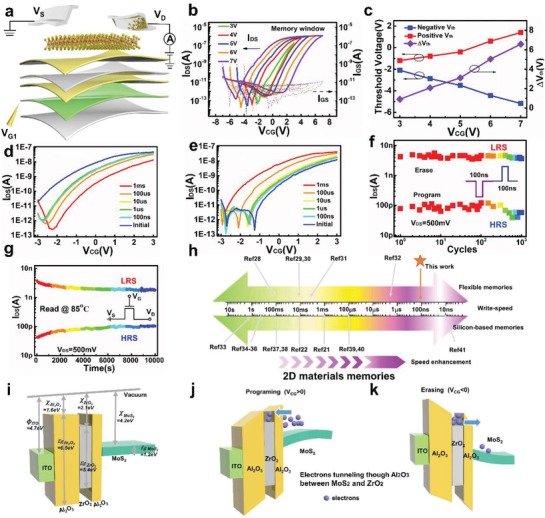
Device characteristics and band diagram of the ultrafast flexible MoS_2_ memory. a) Schematic illustration of device structure for electrical measurement. b) Transfer characteristics of MoS_2_ memory under different *V*_CG_ sweeping ranges (3, 4, 5, 6, and 7 V) and *V*_DS_ = 500 mV. c) Memory window extracted from transfer curve with large hysteresis. d) Program and e) erase speed of device demonstrated using different pulse width of *V*_CG_ (1 ms, 100 µs, 10 µs, 1 µs, 100 ns) after initialization (*V*_CG_ = ±6 V, 1 ms). f) Endurance characteristics of the flexible MoS_2_ memory for 1000 cycles under programming (8.5 V, 100 ns) and erase (−7 V, 100 ns) operations at room temperature, where *V*_DS_ = 500 mV. g) Retention behavior of device after programming and erasing with a 100 ns pulse, where *V*_DS_ = 500 mV. h) Comparison of operation speed of 2D material memories with silicon and flexible substrates. i–k) Schematic band diagram of the MoS_2_ memory under i) flat band state, j) program operation (*V*_CG_ > 0), and k) erase operation(*V*_CG_ < 0). *Φ*, χ, and *E*_g_ represent the work function, the electron affinity, and band gap, respectively.

To further investigate the reliability of the ultrafast device, its endurance and retention characteristics were tested using a 100 ns‐width pulse. By applying sequential programming (8.5 V/100 ns) and erasing (−7 V/100 ns) pulses, the fast‐switched *I*_DS_ was extracted for 10^3^ cycles. As shown in Figure 2f, the on/off ratio was still stable without significant degradation after 10^3^ cycles. The memory window slightly changed and still remained over 10 at 85 °C during the monitoring time (10^4^ s), indicating the potential of our memory in applications using non‐volatile electronics (Figure 2g). The device shows reliable ultrafast operation speed of 100 ns for programming and erasing, which is due to the design of heterostructure‐based insulators (Al_2_O_3_/ZrO_2_/Al_2_O_3_), thin Al_2_O_3_ (≈5 nm) used for tunneling^[^ ^21^, ^27^ ^]^ and the chose MoS_2_ material as channel. The high carrier mobility and steep subthreshold swing make MoS_2_ suitable for charge‐trap memory with high speed charge transport. The comparison between 2D memories and our flexible memory is shown in Figure 2h, where we compare reported memories based on 2D materials.^[^ ^21^, ^22^, ^28^, ^29^, ^30^, ^31^, ^32^, ^33^, ^34^, ^35^, ^36^, ^37^, ^38^, ^39^, ^40^, ^41^ ^]^ It is worth noting that the common programming speeds of 2D material memories are centrally distributed in millisecond timescales while the speed of our flexible memory can be scaled down to 100 ns. This ultrafast flexible memory paves the way for high‐speed and low‐power electronics applications in wearable neuromorphic computing.

To understand the operation mechanism of our memory, the band diagrams in three conditions (flat band, programming and erasing) are shown in Figures 2i–k. The bandgap (*E*_g_) and electronic affinity (χ) of few‐layer MoS_2_ are 1.2 and 4.2 eV. The χ of Al_2_O_3_ and ZrO_2_ is 1.6 and 2.1 eV, respectively. A potential well for electron trapping formed with the sandwiched structure of Al_2_O_3_/ZrO_2_/Al_2_O_3_. During the programming operation (*V*_CG_ > 0), the electrons in MoS_2_ can tunnel through the 5 nm thick Al_2_O_3_ tunneling dielectric to the ZrO_2_ trapping layer based on Fowler–Nordheim tunneling mechanism.^[^ ^42^ ^]^ The accumulated electrons in ZrO_2_ trapping layer could screen the gate electric field effect on the MoS_2_ channel, resulting to a positive shift of the threshold voltage of device (Figure 2j). Erasing phenomenon under negative control gate voltage can be explained as follows. When negative voltage was applied to the gate, the electrons stored in the ZrO_2_ trapping layer could tunnel back to the MoS_2_ channel. Due to the decrease of electrons in ZrO_2_ trapping layer, the screen effect of electrons could be weakened and the threshold voltage of the device could turn to negative position, as shown in Figure 2k. The electron trapping and releasing capabilities result in the reliable memory window of device under different voltages.

The left part of **Figure** **3**a shows the information transmission between pre‐ and post‐synapse in a biological system. The process can be simulated by an electrical pulse applied to our artificial synapse, where the electrical pulse acts as the input spikes and *I*_DS_ as the PSC. EPSC and IPSC were emulated in the artificial synapse by applying separate negative and positive spikes to the ITO gate (Figure 3b,c), respectively. Short‐term synaptic plasticity, including PPF and PPD, is considered critical in decoding temporal vision or auditory information in biological systems, and is triggered by two consecutive presynaptic spikes.^[^ ^43^, ^44^ ^]^ The PPF behavior induced by a pair of relatively negative pre‐spikes is shown in Figure 3d. Figure 3e shows the dependence of the extracted PPF/PPD index (A_2_/A_1_) on the interval time between two pulse (Δ*t*), which was fitted using a double exponential decay function used in biology.^[^ ^45^ ^]^

**Figure 3:**
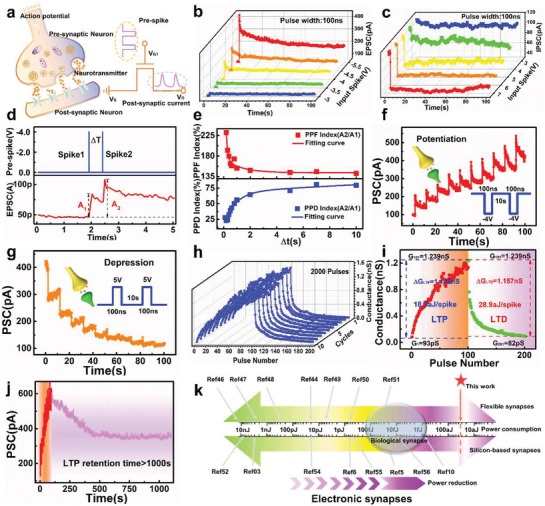
Electrical modulation of ultralow power MoS_2_‐based artificial synapse. a) Schematic of signal transmission from pre‐synapse to post‐synapse in biological synapse and emulation in MoS_2_ device by electrical pulse. b) EPSC triggered by presynaptic pulse with different amplitudes (−3, −3.5, −4, −4.5, and −5 V). The pulse width was fixed at 100 ns. c) IPSC inhibited by different positive pulse (amplitudes: 3, 4, 5, 6, and 7 V) with the same pulse width of 100 ns. d) EPSC generated by a pair of pre‐spikes (−4 V, 100 ns) with an interval of 500 ms. e) PPF and PPD index (A_2_/A_1_ × 100%) as a function of interval time between two continuous pulses, fitted by exponential curves. f) Long‐term potentiation stimulated by a series of pre‐spikes (pulse amplitude = −4 V, pulse width = 100 ns, pulse interval = 10 s). g) Long‐term depression simulation: a series of pre‐spikes (5 V, 100 ns) at 10 s intervals. Inset presents the waveform used for simulation. h) Endurance of potentiation‐depression with 2000 consecutive spikes. i) Ultralow power during LTP and LTD simulation in MoS_2_‐based synapse. Δ*G*_LTP_ is 1.146 nS with 100 negative pulses (−4 V, 100 ns) in LTP, where the average power is 18.3 aJ per spike. j) Long‐term retention behavior after applying 10 pulses (−4 V, 100 ns) for over 1000 s. k) Comparison of power consumption in biological and various electronic synapses. The level of attojoule power consumption is lower than ≈10 fJ of bio‐synapse.

Figure 3f,g show LTP and LTD behaviors with consecutive negative and positive pulses (amplitude = −4 V/5 V, width = 100 ns, number = 10, and interval = 10 s), respectively. The modulated multi‐level states of conductance could be simulated as synaptic weights for neuromorphic computing. The number of applied spikes was increased to 100 and reliability of conductance modulation under 2000 spikes was investigated in Figure 3h. Besides LTP/LTD, spike‐number dependent plasticity, spike‐duration dependent plasticity, and spike‐frequency dependent plasticity were investigated in Figures S4–S6, Supporting Information. Short‐term memory to long‐term memory (STM‐LTM) behaviors indicated higher‐repetition stimulation and could strengthen the synaptic connection in biology (Figure S7, Supporting Information). PSC was also monitored after consecutive pre‐spikes to emulate forgetting behavior (Figure S8, Supporting Information).

The low energy consumption of neuromorphic systems during training and learning is one of the most important advantages of artificial neural networks compared with von Neumann architectures. The energy consumption of our promising synaptic device during LTP and LTD were lower than 30 aJ per spike. MoS_2_ is one kind of typical 2D TMDCs with large bandgap and excellent electrostatic control characteristics, which is idea for low‐power applications. In this work, the standard operating point of *V*_DS_ = 500 mV with *I*_DS_ of pA level, making the power of neuro‐transistor inherently low. The average energy consumption during training can be calculated using the following equation:

(1) $$\bar{W}=\left(V^{2}\times \Delta G\times t_{pulse}\right)/N_{pulse}$$

where *V* is the training voltage, Δ*G* is the delta value of conductance before and after training, *t*_pulse_ is the width of the training pulse and *N*_pulse_ is the number of pulses. Figure 3i shows the calculated energy consumption in LTP (18.3 aJ per spike) and LTD (28.9 aJ per spike). The PSC did not delay back to its initial value after 100 s, indicating the synaptic plasticity of our memory. Long‐term retention (10^3^ s) was measured after ten consecutive negative pulses (pulse width:100 ns, amplitude: −4 V), as shown in Figure 3j. The energy consumption of our flexible MoS_2_‐based device was compared with other synapses, including biological and electronic synapses,^[^ ^5^, ^6^, ^10^, ^44^, ^46^, ^47^, ^48^, ^49^, ^50^, ^51^, ^52^, ^53^, ^54^, ^55^, ^56^ ^]^ and its advantages are demonstrated in Figure 3k. The energy consumption of our synaptic device was orders of magnitude lower than that of biological systems (10 fJ per synaptic event).

Heterosynaptic plasticity, that is, three‐factor learning,^[^ ^57^ ^]^ is an important synaptic synergistically modulated effect induced by neuromodulators in biology, which can be emulated by pre‐, mod‐, and post‐synapse (**Figure** **4**a). An electrical signal was applied to the pre‐terminal while light was used as a signal input of the modulatory terminal to trigger the EPSC in our MoS_2_‐based device. The PSC of the device under illumination at different wavelengths of 250, 300, 350, 400, 450, 500, 550, and 600 nm was monitored at *V*_DS_ = 0.5 V for 15 s (Figure 4b). The light pulse width for each wavelength was fixed at 1 s. Figure 4c further shows the pulse width‐dependent photoresponse of PSC at 350 nm with exposure times of 1 ms, 10 ms, 100 ms, 200 ms, 500 ms, 1 s, 1.5 s, 2 s, 2.5 s, 3 s, 4 s, and 5 s. Besides EPSC, traditional synaptic behaviors including EPSC, STM‐LTM, learning‐forgetting‐relearning rules and LTP/LTD were simulated using a light pulse (λ = 350 nm, pulse width = 1 s). As shown in Figure 4d, the PSC increases with the increase of light pulse number (5, 10, and 50) after the removal of light pulse. Learning‐experience behavior was emulated using two sequences of consecutive light pulses (Figure 4e). After the first 20 light pulses, the spontaneous decay of PSC was monitored for 90 s. The second learning stage required only four pulses to achieve the same current level as in first learning stage, reflecting less time is need for the relearning process, as in the human brain.

**Figure 4:**
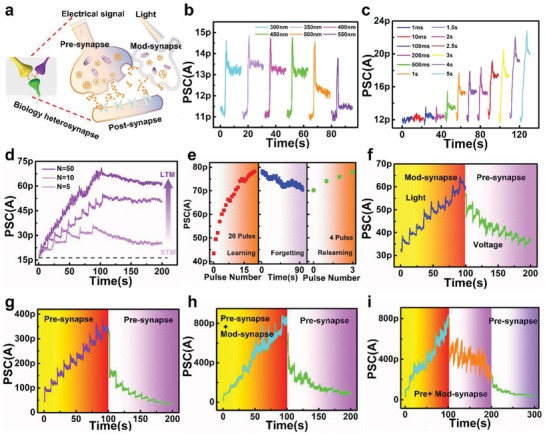
Heterosynaptic plasticity induced by photoelectric synergistic modulation. a) Heterosynapse structure in biological system. The yellow and purple synapses stand for voltage‐controlled pre‐synapse and light‐controlled mod‐synapse, respectively. The green synapse stands for post‐synapse. b) Photoresponse of mod‐synapse under different excitation wavelengths from 250 to 600 nm (exposure time = 1 s) and *V*_DS_ = 500 mV. c) Time‐dependent photocurrents of mod‐synapse induced by light pulses (λ = 350 nm) with different exposure time (1 ms–5 s). d) Change in the post‐synaptic current when the input light pulses were applied at intervals of 20, 10, and 2 s in the initial 100 s. e) Learning, forgetting and relearning behaviors emulated by light‐induced mod‐synapse. f) LTP and LTD stimulated by light pulses (λ = 350 nm, pulse number = 10, exposure time = 1 s) and electrical pulses (amplitude = 5 V, pulse number = 10, pulse width = 100 ns, interval = 10 s) respectively. g) LTP and LTD stimulated by electrical pulses alone. h) Potentiation of heterosynapse controlled by electrical pulses under illumination of light. Then depression was stimulated with electrical pulses alone. i) Electrical pulses induced the LTP and LTD under illumination of light. Further depression was obtained by electrical pulse independently.

By adding light stimulation, complex learning algorithms can be emulated through signal interactions in heterosynapse with two terminal signals, including electrical, and optical spikes. There are four kinds of operation modes for photoelectric synergistic modulation, including pre‐synapse independent modulation, mod‐synapse independent modulation, pre‐, and mod‐synapse synergistic modulation for LTP, and pre‐, and mod‐synapse synergistic modulation for LTD. Figure 4f shows the pre‐synapse independent modulation process. LTP and LTD was induced using light (λ = 350 nm, pulse width = 1 s, pulse number = 10) and electrical pulses (amplitude = 5 V, pulse number = 10, pulse width = 100 ns), respectively. The facilitation and depression behaviors were emulated in electrical modulation mode, corresponding to synaptic plasticity between pre‐ and post‐synapse (Figure 4g). Under illumination of light, electrical and optical pulses were used to emulate a higher order of LTP (Figure 4h), which could be attributed to the effects of illumination (Figure S9, Supporting Information). The depression effect was weakened due to the synergistic modulation of optical and electrical stimulation, while the synaptic plasticity can be returned to its initial state for symmetric modulation through additional electrical spikes, as shown in Figure 4i. To better understand the carrier behaviors when applying light and voltage to device simultaneously, the band diagrams were plotted and described in Figure S10, Supporting Information. All the circuit schematics during measurement of Figure 4 are shown in Figure S11, Supporting Information. Light stimulation can shift the synaptic weights in the positive direction and provide a wider range of weights update in LTP (ratio of facilitation ≈ 228%), offering unique advantages in pattern classification and face recognition based on artificial neuron networks consisting of synaptic devices with multilevel conductance states.

The performance of our flexible MoS_2_‐based device under mechanical stress was investigated (**Figure** **5**a,b). The memory window did not show obvious change under flat and bent states with radius of 10 and 7.5 mm (Δ*V*_th_ = 2.4 V), but for a bending radius *R* = 5 mm (Δ*V*_th_ = 3 V), as shown in Figure 5c. The bent states of the device under different radii including *R* = 5, 7.5, and 10 mm. For *R* = 10 mm, synaptic plasticity including EPSC, IPSC, and LTP/LTD were emulated, providing strong evidence of the stability of our flexible artificial synapse under static bending ( Figure 5d–g). It should be noted that the LTP and LTD behaviors were simulated without any degradation, and even some enhancement was observed (Figure 5f,g), demonstrating the reliability and feasibility of our flexible artificial synaptic device.

**Figure 5:**
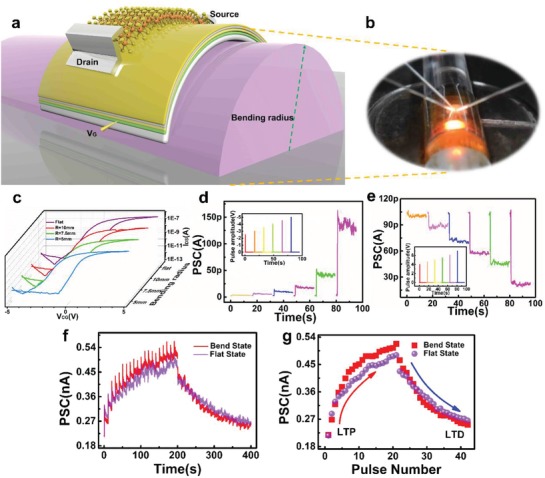
Flexibility of MoS_2_‐based artificial synapse. a) Schematic and b) optical image of flexible device measurement in curved state. The thickness of PET is 175 µm c) Transfer characteristics of device under flat state and with a radius of curvature of 10, 7.5, and 5 mm. d,e) Amplitude‐dependent d) EPSC and e) IPSC of curved device (*R* = 10 mm) at different electrical pulses with width of 100 ns. Inset presents the pulse amplitude applied to device. f) EPSC and IPSC stimulated by consecutive electrical pulses (amplitude = −4 V/5 V, pulse number = 20, pulse width = 100 ns) with interval of 10 s under flat state and curved state (*R* = 10 mm). g) Controlled post‐synaptic current as a function of pre‐synaptic pulse number in a flat states and curved state (*R* = 10 mm).

In this work, a MoS_2_‐based artificial heterosynaptic device with photoelectric synergistic modulation for wearable applications was demonstrated. The devices exhibited reliable memory characteristics with an ultrahigh on/off ratio of 10^6^, stable switching cycles of 10^3^ and excellent storage time of 10^4^ s. To emulate biological synapses for neuromorphic computing, the operating speed and energy consumption achieved were 100 ns and ≈30 aJ per spike, respectively. The implementation of complex neural behaviors requires neuromodulation based on multi‐terminal synapses, which are usually ignored by most studies. Light, as a potential modulation signal, can be used for mimicking traditional synaptic plasticity. By adding additional optical stimulation as the modulatory synapse, heterosynaptic plasticity was emulated in our flexible device. Comparing with separate electrical modulation, photoelectric synergistic modulation can strengthen LTP for higher order correlations (ratio of facilitation ≈ 228%) and weaken the LTD effect by 25%. The coexistence of optical and electrical modulation in our multi‐terminal device opens a new path for complex biological activity simulation and neuromorphic computing system design.

## Experimental Section

##### Device Fabrication

The ITO‐coated PET was used as a substrate with gold mark by sputtering. A low‐temperature growth process by ALD at 130° was used to prepare the dielectric layer Al_2_O_3_/ZrO_2_/Al_2_O_3_. The MoS_2_ flake was exfoliated from bulk crystals onto the PET substrate directly. The source and drain were patterned using electron‐beam lithography and formed by Ti(15 nm)/Pt(50 nm) using a sputtering system.

##### Device Characterizations

Electrical measurements were carried out using a semiconductor parameter analyzer (Agilent B1500A) under dark conditions. Electrical pulses were produced using a semiconductor pulse generator unit (SPGU) module. Light pulses with tunable wavelength were produced using a xenon lamp system. The surface morphologies of the device were measured using AFM in tapping mode. Raman spectroscopy was obtained using a Horiba XploRA Raman spectrometer with a 532 nm excitation source. All the electrical and optical measurements were performed in air atmosphere of clean room with temperature of ≈23 °C and humidity ≈45%.

## Conflict of Interest

The authors declare no conflict of interest.

## Supporting information

Supporting Information
