# Supplementary material for: Ultralow Power Wearable Heterosynapse with Photoelectric Synergistic Modulation
Source: Adv Sci (Weinh). 2020 Mar 16;7(8):1903480. doi: 10.1002/advs.201903480 (PMC7175259; doi:10.1002/advs.201903480)
Supplement: Supplementary file 1 — Supporting Information [file ADVS-7-1903480-s001.pdf]

Copyright WILEY-VCH Verlag GmbH & Co. KGaA, 69469 Weinheim, Germany, 2020.

ADVANCED SCIENCE Open Access

Supporting Information

for *Adv. Sci.,* DOI: 10.1002/advs.201903480

Ultralow Power Wearable Heterosynapse with Photoelectric Synergistic Modulation

Tian-Yu Wang, Jia-Lin Meng, Zhen-Yu He, Lin Chen, Hao Zhu, Qing-Qing Sun,* Shi-Jin Ding, Peng Zhou, and David Wei Zhang*

Supportting Information

Ultralow Power Wearable Heterosynapse with Photoelectric Synergistic Modulation

Tian-Yu Wang[1], Jia-Lin Meng[1], Zhen-Yu He[1], Lin Chen[1]*, Hao Zhu[1], Qing-Qing Sun[1]*, Shi-Jin Ding[1], Peng Zhou[1], and David Wei Zhang[1]

[1]State Key Laboratory of ASIC and System, School of Microelectronics, Fudan University, Shanghai 200433, China

*Correspondence and requests for materials should be addressed to L.C. and Q.Q.S. (E-mail : linchen@fudan.edu.cn; qqsun@fudan.edu.cn;)

S1. Fabrication process of MoS_2-based flexible synaptic device.

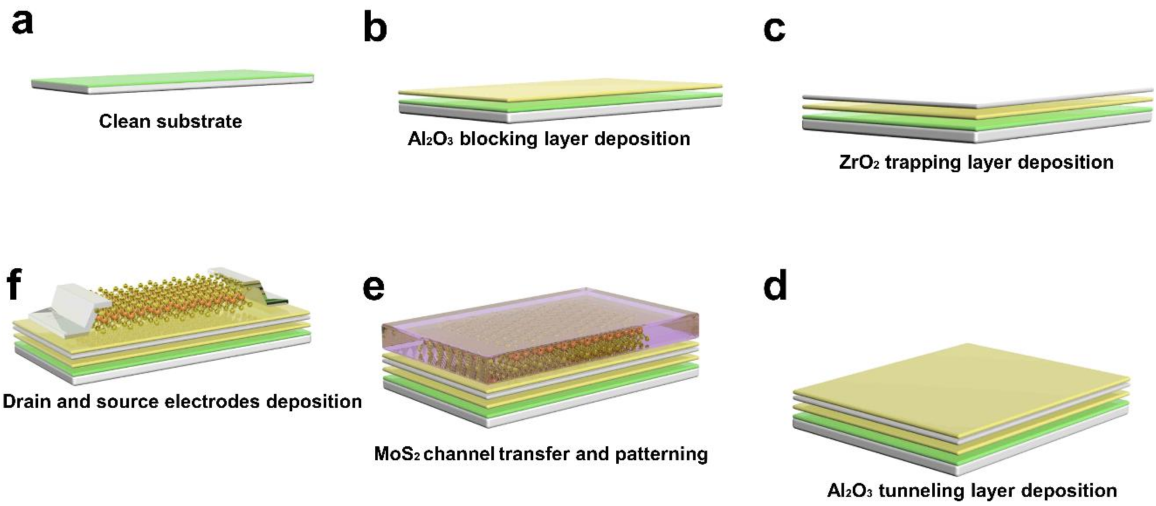

Figure S1. The flexible 2D device fabrication process flow. (a) A flexible ITO coated PET substrate with gold mark was cleaned with acetone, isopropyl alcohol (IPA) and deionized water. (b) Blocking dielectric layer Al_2O_3 of 15.6nm is deposited by atomic layer deposition (ALD) at 130℃. (c) High-k dielectric ZrO_2 trapping layer of 11.2nm and (d) tunneling layer Al_2O_3 of 4.8nm are deposited by the same method. (e) About 17nm of MoS_2 are exfoliated from the bulk material and transferred to flexible substrate. Source and drain is pre-formed by electron beam lithography (EBL). (f) Ti/Pt (15nm/50nm) is deposited by sputtering to form electrode.

S2. EDX line scanning spectrum and mapping image.

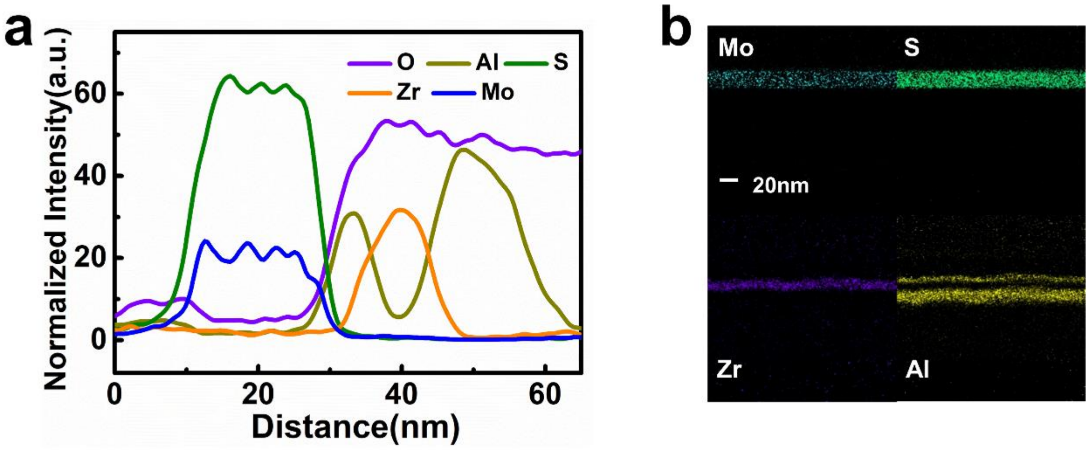

Figure S2. TEM-EDX analysis of channel and oxide layer. (a) EDX line scanning profiles from MoS_2 channel to tunneling, trapping and blocking layers. (b) EDX elements map of Mo, S, Zr and Al, indicating distribution of elements in MoS_2 channel and oxide dielectric layers. The scale bar is 20nm.

S3. Memory characteristics in electrical mode.

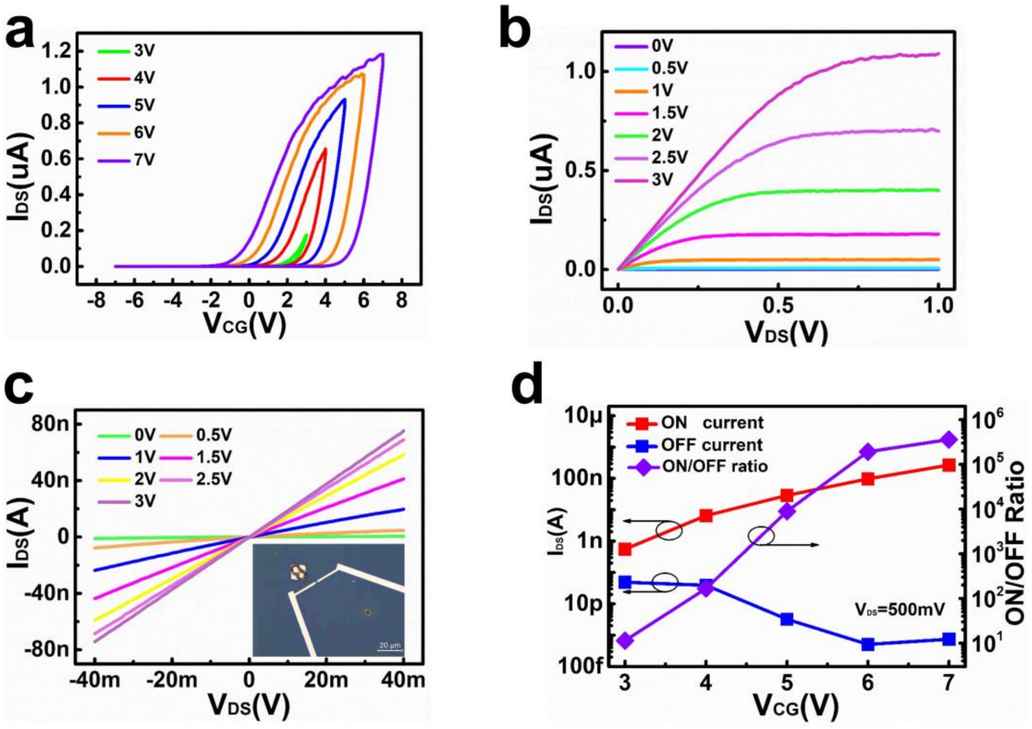

Figure S3. Transfer and output characteristics of the MoS_2-based memory. (a) Dual sweep I_{DS}-V_{CG} curve of device with memory window. Different V_{CG} of 3V, 4V, 5V, 6V and 7V were applied to the back gate while the I_{DS} was monitored, where V_{DS}=500mV. The memory window increased with the increment of V_{CG}. (b) Output characteristics of I_{DS}-V_{DS} at different V_{CG} (0V, 0.5V, 1V, 1.5V, 2V, 2.5V and 3V). (c) I_{DS}-V_{DS} output curves across the MoS_2 flake recorded in the small scan range of V_{DS} (-40mV to 40mV). The results demonstrated Ohmic contacts based on the results the I_{DS} are linearly dependent on V_{DS}. Inset shows the microscope image of device with channel length of 2um and width of 1um. (d) The on/off ratio extracted from transfer curve with hysteresis.

S4. The spike-number dependent plasticity (SNDP) of artificial synapse.

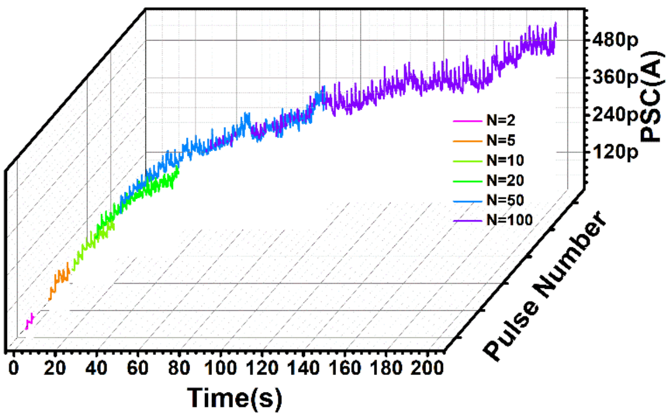

Figure S4. The synaptic plasticity of SNDP in our device. Long-term potentiation is one of important synaptic plasticity, which could be modulated by the input pre-spikes. By applying pre-spikes (pulse amplitude=-4V, pulse duration=100ns, pulse frequency=0.5HZ) to artificial synaptic device, the PSC under different pulse number were recorded of 68pA (N=2), 122pA (N=5), 175pA (N=10), 269pA (N=20), 425pA (N=50) and 535pA (N=100). With the increase of applied pulse number from 2 to 100, the synaptic weights would increase obviously in our artificial synapse.

S5. The spike-duration dependent plasticity (SDDP) of artificial synapse.

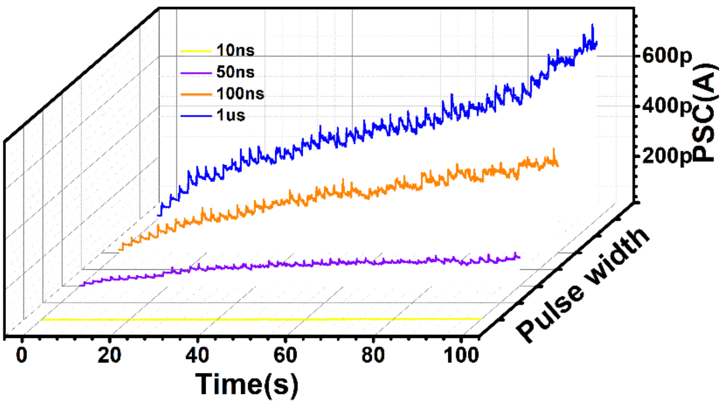

Figure S5. The synaptic plasticity of SDDP in our device. By applying consecutive pre-spikes (pulse amplitude=-4V, pulse number=50, pulse frequency=0.5HZ) to the gate terminal of artificial synapse, the PSC under different pulse duration were recorded of 20pA (pulse duration=10ns), 129pA (pulse duration=50ns), 362pA (pulse duration=100ns) and 726pA (pulse duration=1μs). With the increase of pulse duration from 10ns to 1us, the synaptic weights would be enhanced in our artificial synapse.

S6. The spike-frequency dependent plasticity (SFDP) of artificial synapse.

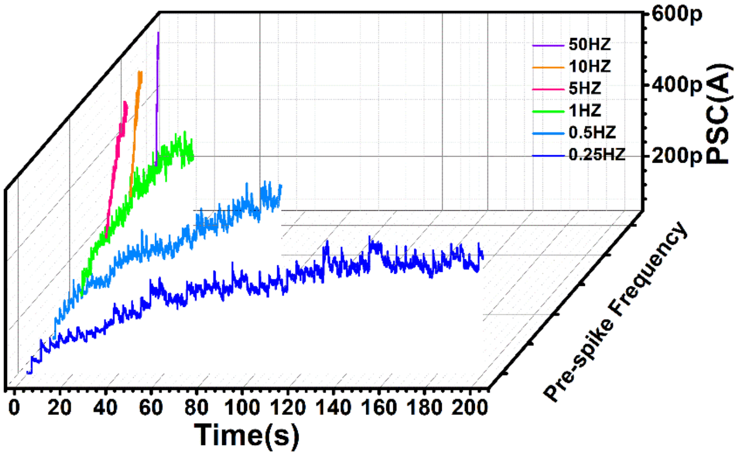

Figure S6. The synaptic plasticity of SFDP in our device. Long-term potentiation could be triggered by input pulses (pulse amplitude=-4V, pulse number=50, pulse duration=100ns) applied to pre-synapse. To investigate the influence of pulse frequency in LTP, the PSC under different pulse frequency were recorded of 377pA (pulse frequency=0.25HZ), 494pA (pulse frequency=0.5HZ), 503pA (pulse frequency=1HZ), 544pA (pulse frequency=5HZ), 547pA (pulse frequency=10HZ) and 590pA (pulse frequency=50HZ). With the increase of pre-spike frequency from 0.25HZ to 50HZ, the correlations between pre- and post-synapse would be strengthened.

S7. The short-term memory (STM) to long-term memory(LTM).

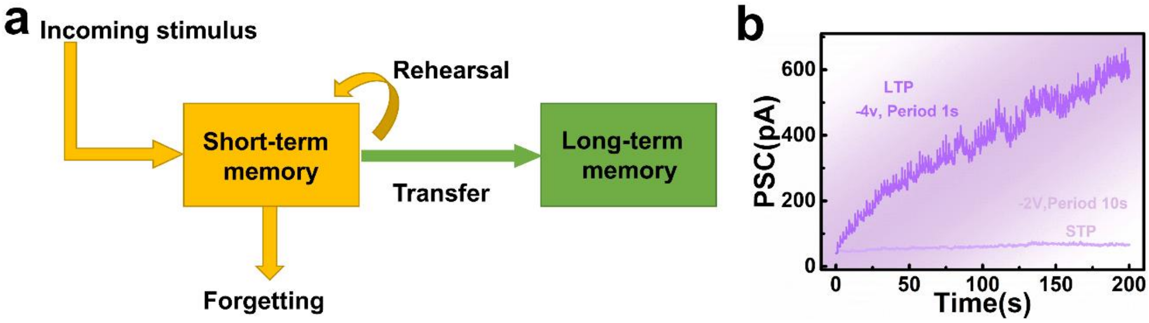

Figure S7. Mode and simulation of STM and LTM phenomena. (a) Illustration of STM to LTM transition in human brain by Atkinson and Shiffrin[1]. The incoming stimulus was received by STM and some information could retain for a short time with the process of forgetting. By repeated rehearsals and learning, some information was transferred to LTM and last for a long time. The result indicated that frequent and intense stimulations could trigger LTM in biology even artificial synaptic device. (b) The phenomena of temporary STM to permanent LTM was emulated by our MoS_2-based artificial synapse to verify the rehearsal effect. Different times intervals (1s and 10s) were used in pre-spikes and the increased PSC was associated with the consequent memory transition.

S8. The phenomena of forgetting in human brain.

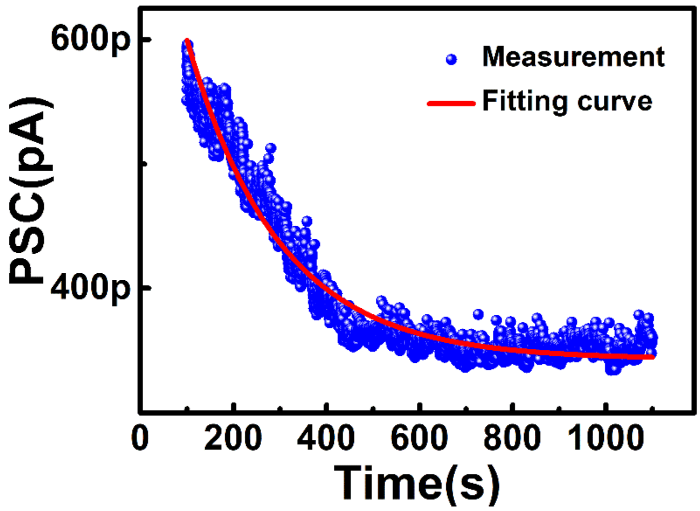

Figure S8. Forgetting curve and fitted line using exponential relaxation model. The PSC was monitored after 200 consecutive pulses (pulse amplitude=-4V, pulse duration= 100ns, pulse frequency= 1HZ), which emulated the forgetting function in biology. The forgetting curve could well fitted by formula known as the Kohlrausch law[2]:

$$I(t) = I_0 + A\exp(-t/\tau)$$

where *I(t)* is the relaxation function and I_0 is the stabilized current. A is the prefactor and τ is the characteristic relaxation time. In our synaptic device, the I_0 is 343pA and τ is 198s, indicating the current of final state and speed of relaxation.

S9. The PSC of device under light and dark condition.

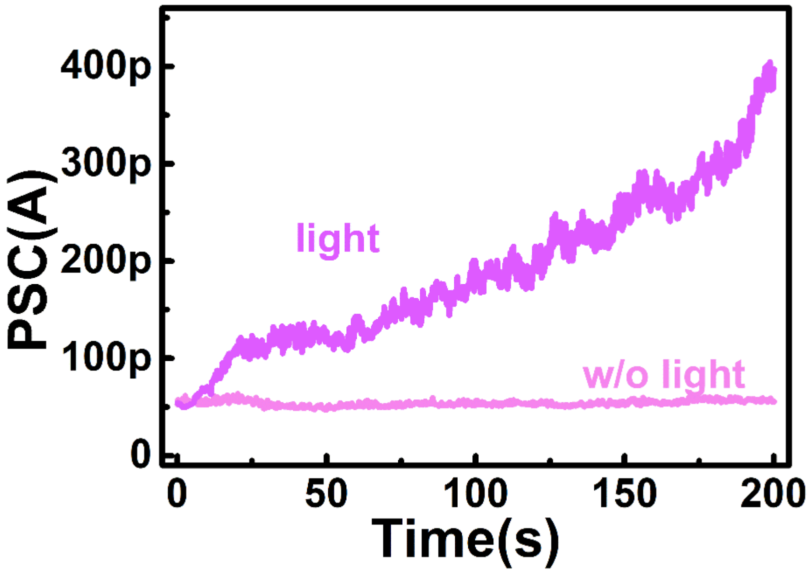

Figure S9. The PSC would increase when device is exposed to light. Under illumination of wavelength of 350nm, the PSC would achieve ~400pA, indicating the potential of MoS_2-based heterosynapse used for multi-terminal modulation by optical and electrical signals. The light stimulation could result to a higher order of LTP.

S10. The band diagram of the device under voltage and light simultaneously.

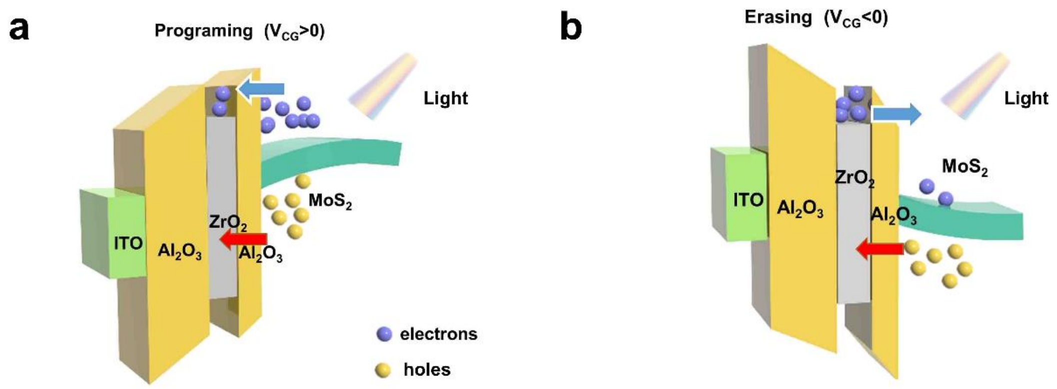

Figure S10. There are two possible scenarios when applying control voltage and light simultaneously to synaptic transistor, including a) illumination combined with positive voltage and b) illumination combined with negative voltage, respectively. a) When applying light and positive voltage simultaneously to the device, the positive voltage could result to the electrons of MoS_2 tunneling to ZrO_2 trapping layer. While the photoexcited holes in the valence band of MoS_2 could overcome the energy barrier between the channel of MoS_2 and the tunneling layer of Al_2O_3 and trapped in ZrO_2 layer, which could combine with some electrons in ZrO_2 layer and weaken the positive voltage effect. b) When applying light and negative voltage simultaneously to the synaptic transistor, the effect of voltage and light are consistent. Under negative voltage, the electrons stored in the ZrO_2 trapping layer could tunnel back to the MoS2 channel and cause the threshold voltage shifting to the negative direction. With light illumination, the photoexcited holes in the valence band of MoS_2 could tunnel to ZrO_2 layer, resulting to a negative shift of the threshold voltage. The light and negative voltage are both helpful to shift the threshold voltage to the negative direction.

S11. Circuit schematics of the heterosynapse.

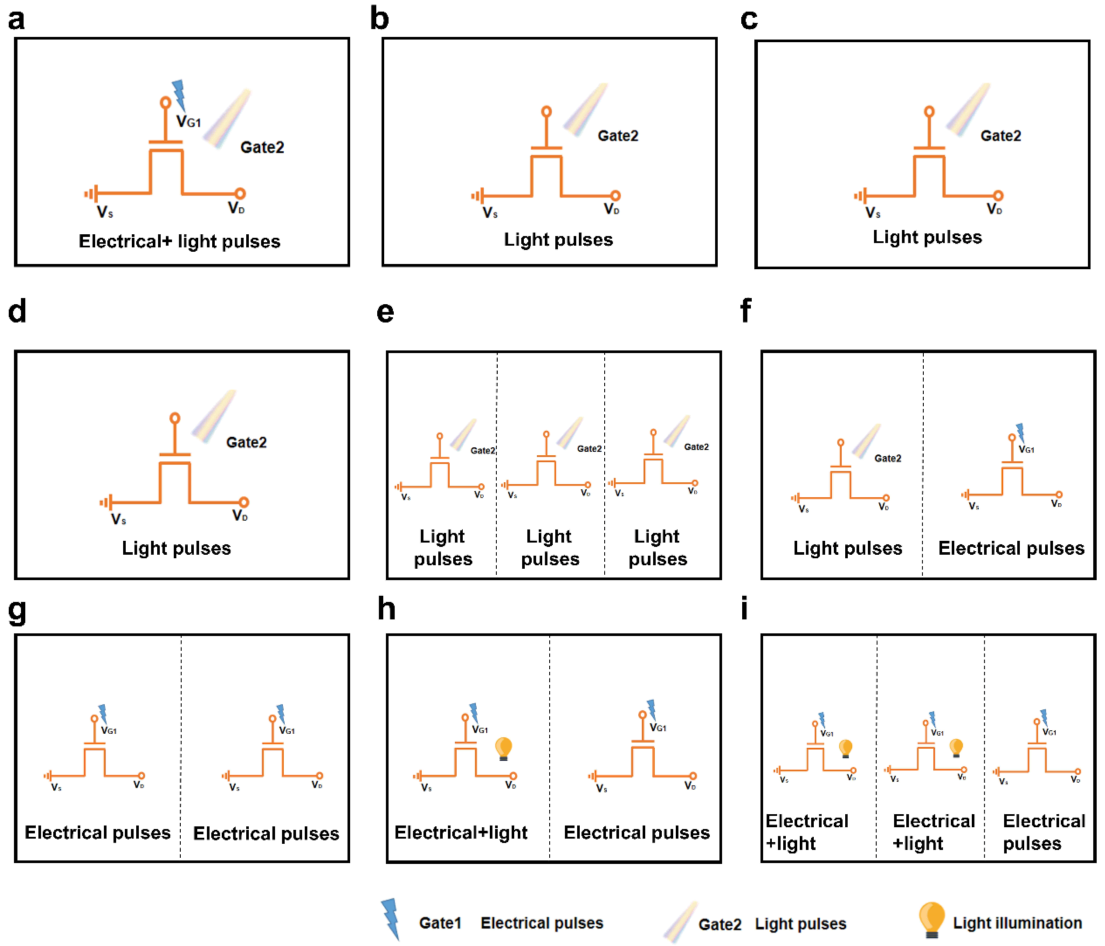

Figure S11. Circuit schematics of the device under the various operating conditions for heterosynaptic plasticity, corresponding to "Figure 4" in manuscript. a) Heterosynapse inspired by electrical and light pulses. b) Modulation of mod-synapse under different light pulses. c) Time-dependent mod-synaptic plsticity. d) Short-term memory to long-term memory simulated by light pulses. e) Learning, forgetting and relearning behaviors emulated by light pulses. f) Light pulses for LTP and electrical pulses for LTD, respectively. g) Electrical pulses simlutate LTP and LTD without light. h) LTP simulated by electrical pulses under illumination of light. LTD stimulated with electrical pulses without light. i) LTP and LTD emulated by electrical pulses under illumination. Depression simulated by electrical pulse alone.

Reference

[1] R. C. Atkinson, R. M. Shiffrin, in Psychology of Learning and Motivation, Vol.2 (Eds: K. W. Spence, J. T. Spence), Academic Press, **1968**, 89.

[2] T. Chang, S.-H. Jo, W. Lu, ACS Nano **2011**, 5, 7669.
